# Is Adjuvant Cellular Immunotherapy Essential after TACE-Predominant Minimally-Invasive Treatment for Hepatocellular Carcinoma? A Systematic Meta-Analysis of Studies Including 1774 Patients

**DOI:** 10.1371/journal.pone.0168798

**Published:** 2016-12-22

**Authors:** Min Ding, Ying Wang, Jiachang Chi, Tao Wang, Xiaoyin Tang, Dan Cui, Qijun Qian, Bo Zhai

**Affiliations:** 1 Department of Interventional Oncology, Renji Hospital, School of Medicine, Shanghai Jiaotong University, Shanghai, China; 2 Laboratory of Gene and Viral Therapy, Eastern Hepatobiliary Surgery Hospital, the Second Military Medical University of Chinese PLA, Shanghai, China; The First Affiliated Hospital of Nanjing Medical University, CHINA

## Abstract

**Purpose:**

Cellular immunotherapy has appeared to be a promising modality for the treatment of malignant tumor. The objective of this study was to evaluate the efficacy of cellular immunotherapy combined with minimally invasive therapy.

**Methods:**

We searched PubMed, Web of Science and The Cochrane Library through March 2016 for relevant studies. Short-term efficacy (the disease control rate, the control rate of quality life and the AFP descent rate) and long-term efficacy (overall survival (OS) and progression-free survival (PFS) rate) were compared as the major outcome measures. The meta-analysis was performed using Review Manager 5.3.

**Results:**

A total of 1174 references in 3 databases were found of which 19 individual studies with 1774 HCC patients enrolled in this meta-analysis. Meta-analysis results showed that cellular immunotherapy combined with minimally-invasive treatment significantly improved the measures of short-term response (the disease control rate (OR = 5.91, P = 0.007), the control rate of quality lift (OR = 3.38, P = 0.003) and the AFP descent rate (OR = 4.48, P = 0.02)). Also higher 6-month PFS (OR = 2.78, P = 0.05), ≥12-month PFS (OR = 3.56, P<0.00001) rate and 6-month OS (OR = 2.81, P = 0.0009), 12-month OS (OR = 3.05, P<0.00001) and 24-month OS (OR = 3.52, P<0.0001) rate were observed in patients undergoing cellular immunotherapy.

**Conclusions:**

This meta-analysis suggested that cellular immunotherapy is a feasible adjuvant treatment that could be beneficial for the improvement of the clinical outcomes for hepatocellular carcinoma (HCC) patients after minimally invasive treatment, including short-term response and long-term survival.

## Introduction

Hepatocellular carcinoma (HCC) is the most common type of hepatobiliary cancer, the fifth common malignant cancer and the third most cause of cancer-related deaths worldwide [1]. The growth of annual incidence and mortality of HCC is higher in Asia countries accounting for almost 80%, especially in China [2]. Currently, the recurrence rate of HCC was still high after conventional radical resection therapies. Moreover, not all patients may benefit from hepatectomy because of the high incidence of complications due to chronic liver disease or with intermediate-stage HCC [3,4]. Minimally-invasive treatment has been widely used for patients with unresectable HCC. Transcatheter arterial chemoembolization (TACE) has been found to be an effective method to reduce HCC tumor size and improve overall survival (OS) [5,6]. Also, some studies showed that radiofrequency ablation (RFA) and microwave ablation (MWA) could increase tumor necrosis and prolong survival time for HCC [7,8]. However, there were still some limitations existed in long-term prognosis. The recurrence and metastasis after treatment with the TACE-predominant minimally-invasive treatment were still frequent [9]. The prognosis of HCC remained dismal with a low level of survival (5-year survival rate less than 5%) in patients with advanced HCC at diagnosis [10].

In recent decades, cellular immunotherapy has emerged as a promising strategy for cancer treatment [11,12]. It has been reported that cellular immunotherapy could strengthen the immune state and afford a potential value in enhancing the therapeutic outcome [13], although it has not been considered as a standard therapy for HCC. Several studies and meta-analysis had revealed that the combination of cellular immunotherapy of conventional therapies was more effective [14,15]. However, these analysis were about the combination of specific cellular immunotherapy and specific interventional therapy. There were no studies systematically analyzed whether cellular immunotherapy either CIK or DC-CIK was needed after different interventional therapy. The efficacy of cellular immunotherapy still remains controversial, especially in prolonging progression-free and overall survival [16,17].

Therefore, we summarized the trials of cellular immunotherapy, including CIK and DC-CIK, combined with TACE-predominant minimally-invasive treatment for HCC to systematically evaluate the efficacy of cellular immunotherapy.

## Materials and Methods

### Search strategy for identification of studies

A comprehensive literature search process was conducted in Pubmed, Web of Science and The Cochrane Library, based on combinations of the following keywords: [hepatocellular carcinoma OR liver neoplasms OR liver cancer] AND [(lymphokine-activated killer OR LAK) OR (cytokine induced killer cell OR CIK) OR (tumor infiltrating lymphocyte OR TIL) OR (cytotoxic T lymphocyte OR CTL) OR (dendritic cell OR DC) OR (natural killer cell OR NK)]. We searched these keywords in Title/Abstract of literatures about human between January 2006 and March 2016. Studies were not limited to language.

### Inclusion and exclusion criteria for studies in this meta-analysis

In this systematic analysis, literatures were included that met the following criteria: (i) reported clinical outcomes of cellular immunotherapy for HCC; (ii) the case-control study design; (iii) provided enough information to calculate the odds ratio for short term efficacy, PFS or OS.

The exclusion criteria were as follows: (i) reviews, case reports, in vitro experiments, animal models, other diseases or other treatments; (ii) studies without appropriate control groups or enrolled patients less than 15.

### Data extraction and quality assessment

All candidate literatures were reviewed and retrieved by two independent authors (Min Ding and Ying Wang) from these 3 databases, discussed and arrived at a consensus with a third author if disagreement occurred, and extracted data. Articles that could not be categorized according to title or abstract alone were retrieved for full-text review.

The following information was extracted from each included article: authors, year of publication, tumor characteristics, case numbers, regimens, immune cell regimens and culture of immune cells. Two independent authors (Min Ding and Ying Wang) evaluated each study for risk of bias and applicability by using The Quality Assessment of Diagnostic Accuracy Studies-2 (QUADAS-2) tool [18]. The QUADAS-2 contained four key domains covering patient selection, index test, reference standard, and flow and timing. The “low risk of bias”, “unclear risk of bias” or “high risk of bias” for each article was attributed in Cochrane reviews of interventions. Any disagreement between two authors regarding quality assessment was resolved through discussion with the third author.

### Statistical methods

The analysis was carried out using pair-wise comparison between immunotherapy arms and control arms. Review Manager 5.3 [6] was used to conduct meta-analysis and calculated odds ratio (OR) for the disease control rate, the control rate of quality life and the AFP descent rate to reflect short-term efficacy, and 6-, 12-, 24-month PFS and OS to reflect long-term treatment effects across studies. The number of events evaluated in each arm was utilized to calculate the pooled OR with 95% confidence intervals (CI) combined the Mantel-Haenszel statistical method with random effects. The Cochran’s Q test (chi-square statistic; x^2^) was applied to evaluate the heterogeneity among studies. We considered inconsistency index (I^2^) [19] with 25%, 50%, and 75% as representing the evidence of low, moderate, and high heterogeneity, respectively. Publication bias was assessed with Peters test using Meta R package. A p-value of no greater than 0.05 is considered to be statistically significant.

## Results

### Study characteristics

**Fig 1:**
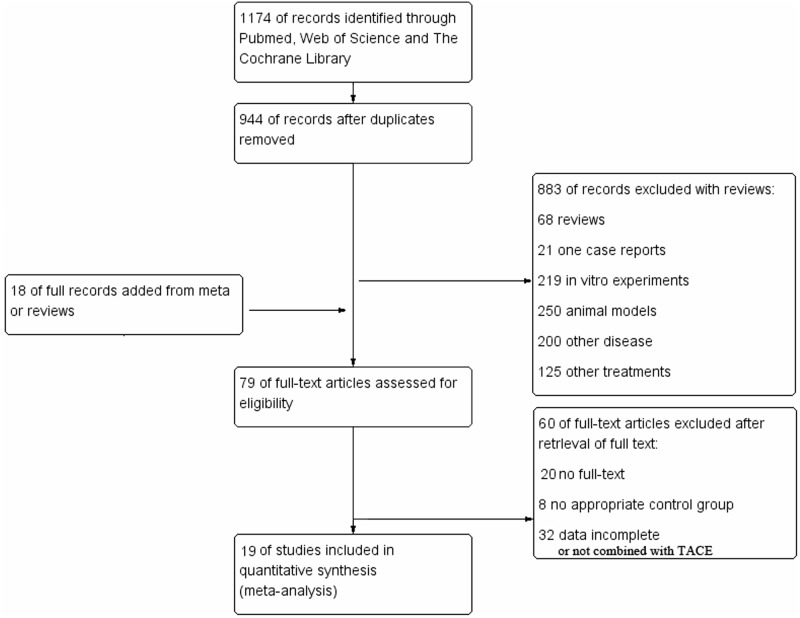
Flow chart of the included studies.

A search strategy in databases of PubMed, Web of science and The Cochrane Library was applied to find a total of 1174 citations, of which 230 were discarded as they were duplicates. After title and abstract screening, 883 articles were excluded for the following reasons: 68 were reviews, 219 were in vitro experiments, 250 used animal models, 21 were a case report, 200 studied other diseases and 125 used other treatments. Additional 18 literatures were supplemented from meta-analysis studies or reviews. In the third step of the eligibility and detailed assessment, 79 full-texts were retrieved, and 60 articles without a control group, full-text, complete clinical data or not combined with TACE-predominant minimally-invasive treatment were excluded (Fig 1). Finally, we included 19 studies with 1774 HCC patients in quantitative synthesis for meta-analysis.

The characteristics of the populations presented in these reports were summarized in Table 1. Overall, 13 studies addressed passive immunotherapeutic approaches including cytokine-induced killer cells (CIKs) [20–32]. Other six studies administrated DC-CIK cells [33–38]. Tumor characteristics and regimens were reported in all studies.

**Table 1:** Clinical information of the trials for the immunotherapy.

Reference	Tumor characteristics	Patients No (control)	Regimens (per arm)	Immune cell regimens	Culture of immune cell
**Zhao 2006**	HCC	33(31)	TACE+RFA+CIK,TACE+RFA	1.1–1.5×10^10^/course	CM,CD3MeAb,IL-2,INF-r,IL-1a.
**Hao 2006**	HCC	21(46)	TACE+CIK,TACE	1–5×10^10^/course	CM, IFN-r, CD3McAb, IL-1a, IL-2.
**Zhang 2006**	HCC	52(92)	TACE+CIK/TACE+PEI+CIK,TACE/TACE+PEI	1.0–1.2×10^10^/course	CM, IFN-r, CD3McAb, IL-1a, IL-2.
**Shi 2007**	HCC	38(214)	TACE+CIK,TACE/TACE+PEI	1.0–1.2×10^10^/course	CM, IFN-r, CD3McAb, IL-1a, IL-2.
**Huang 2007**	Primary HCC	55(30)	TACE+RFA+CIK,TACE+RFA		
**Weng 2008**	HCC	45(40)	TACE+RFA+CIK,TACE+RFA	1.0–2.0×10^10^/course	CM,IFN-r,CD3 McAb,IL-1a,IL-2.
**Hao 2010**	HCC	74(72)	TACE+CIK,TACE	1–5×10^10^/course	Serum-free culture medium,IFN-r,CD3 McAb,IL-1a,IL-2.
**Pan 2010**	HCC	42(39)	TACE+RFA+CIK,TACE+RFA	Once every week, at least 4 infusions, more than 1×10^10^ cells per course.	
**Wu 2012**	Primary HCC	32(38)	TACE+DC-CIK,TACE		
**Wang 2012**	HCC	38(38)	TACE+RFA+CIK,TACE+RFA	Once every twice weeks, at least 3 infusions,1–1.5×10^10^ cells per course.	IFN-r,CD3 McAb, IL-1a,IL-2.
**He 2012**	Primary liver cancer	60(58)	TACE+CIK,TACE	1 week after TACE	
**Huang 2013**	HCC	85(89)	TACE+RFA+CIK,TACE+RFA	2 weeks after sequential TACE and RFA, the median successive number of CIK cell infusions was 9 (range, 4–25).	IFN-r, CD3-McAb, IL-2, IL-1a.
**Deng 2013**	HCC	20(21)	TACE+RFA+CIK,TACE+RFA		
**Tong 2013**	Primary liver cancer	20(18)	TACE+ CIK,TACE	1–5×10^10^/course	IFN-r, CD3-McAb, IL-2
**Xu 2013**	Large HCC	40(40)	TACE+PMCT+DC-CIK,TACE+PMCT		
**Guo 2014**	Primary liver cancer	30(38)	TACE+DC-CIK,TACE	1.0×10^10^/course	AIM-V serum-free culture medium, IFN-r,rhIL-2,CD3 McAb,IL-1,GM-CSF,rhIL-4,TNF-a.
**Zhang 2014**	liver cancer	41(44)	TACE+RFA+DC-CIK,TACE+RFA	6 times 7 days after TACE and RFA, and the number of DC-CIK cells was above 1.0×10^10^	
**Liu 2014**	Advanced HCC	23(17)	TACE+DC-CIK,TACE		
**Cheng 2014**	liver cancer	32(28)	TACE+DC-CIK,TACE	4 infusions of DC-CIK cells	IFN-r, CD3-McAb, IL-2, IL-1a, GM-CSF, IL-4, HSP.

### Quality assessment

The 19 included papers comprised 11 randomized controlled studies [20–22,26,28,29,32,34,35,37,38], 1 studies without describing the randomization method [25] and 7 retrospective studies with a matched-pair control group [23,24,27,30,31,33,36]. It contained eleven (57.89%) with all low risk of bias, one (5.26%) with unclear risk and seven (36.84%) with high risk of bias in random sequence generation, allocation concealment, blinding of participants and personnel and blinding of outcome assessment items, as was shown in S1 Fig.

### Short-term efficacy

**Fig 2:**
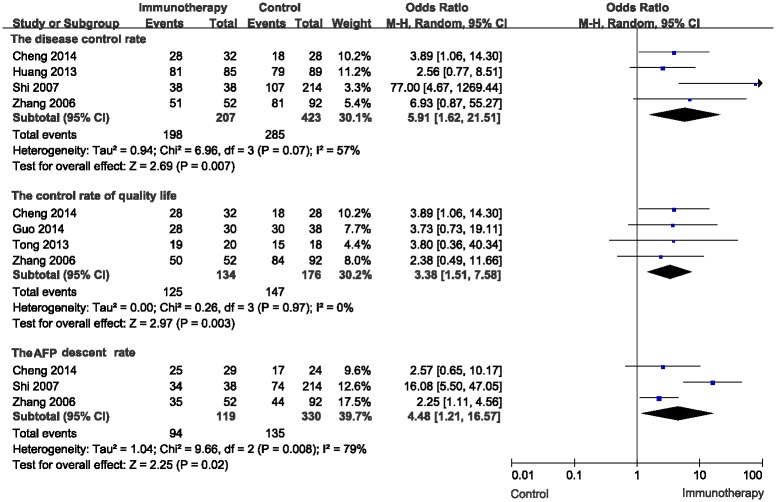
Comparison of short-term efficacy between the patients undergoing cellular immunotherapy or not by using the random effects model (Mantel-Haenszel method).

The disease control rate, the control rate of quality life and the AFP descent rate were used to evaluate the short-term efficacy of the cellular immunotherapy. For the disease control rate, the meta-analysis for three CIK studies and one DC-CIK studies showed a significant advantage of the combination therapy (OR = 5.91, 95% CI: 1.62–21.51, P = 0.007). And the heterogeneity was not statistically significant with P value equal to 0.07 (Fig 2A). Four studies, including two CIK studies and two DC-CIK studies were used to evaluate the control rate of quality life in the meta-analysis. The control rate of quality life was significantly higher in patients with cellular immunotherapy compared to without (OR = 3.38, 95% CI: 1.51–7.58, P = 0.003). Also, there was no heterogeneity among the studies (I^2^ = 0%, P = 0.97) (Fig 2B). For the AFP descent rate, the meta-analysis of three studies (two CIK studies and one DC-CIK studies) showed the combination therapy significantly descent the AFP level (OR = 4.48, 95% CI: 1.21–16.57, P = 0.02) (Fig 2B).

### Long-term efficacy

To evaluate the long-term efficacy of the combination of cellular immunotherapy and TACE-predominant minimally-invasive treatment, we performed subgroup analysis to conduct meta-analysis according to 6-month, 12-month and 24-month progression free survival and overall survival.

#### Progression free survival

For total effect, progression free survival rate was significantly higher in HCC patients undergoing cellular immunotherapy, compared to patients in control groups (OR = 3.46; 95% CI: 2.37–5.06, P<0.00001). All the Cochran’s Q-test resulted in a P value of 0.20, and the corresponding I^2^ was 24%, indicating that the degree of variability was basically consistent in each result of study.

**Fig 3:**
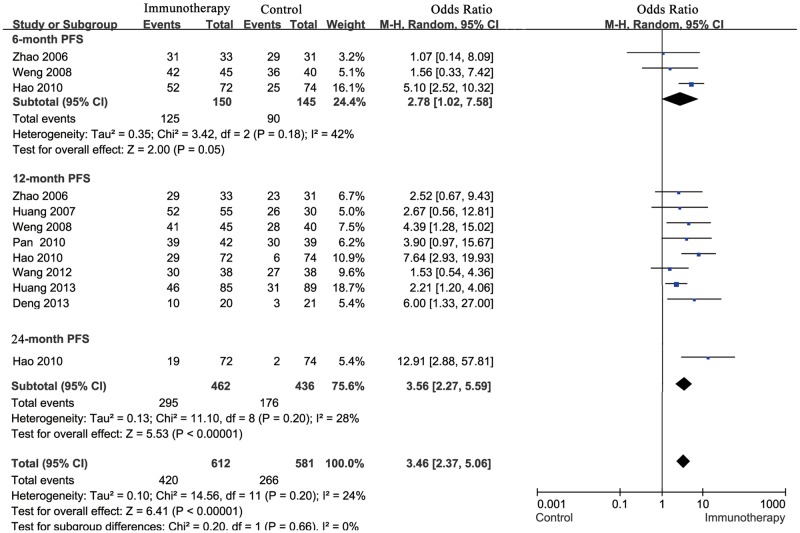
Comparison of 6, ≥12-month PFS between the patients who received cellular immunotherapy or not by using the random effects model (Mantel-Haenszel method).

In subgroup meta-analysis of studies on 6-, > = 12-month progression free survival, patients in cellular immunotherapy treatment group had significantly higher PFS than those in control group, accordingly (6-month: OR = 2.78; 95% CI: 1.02–7.58, P = 0.05; > = 12-month: OR = 3.56; 95% CI: 2.27–5.59, P<0.00001) (Fig 3).

#### Overall survival

**Fig 4:**
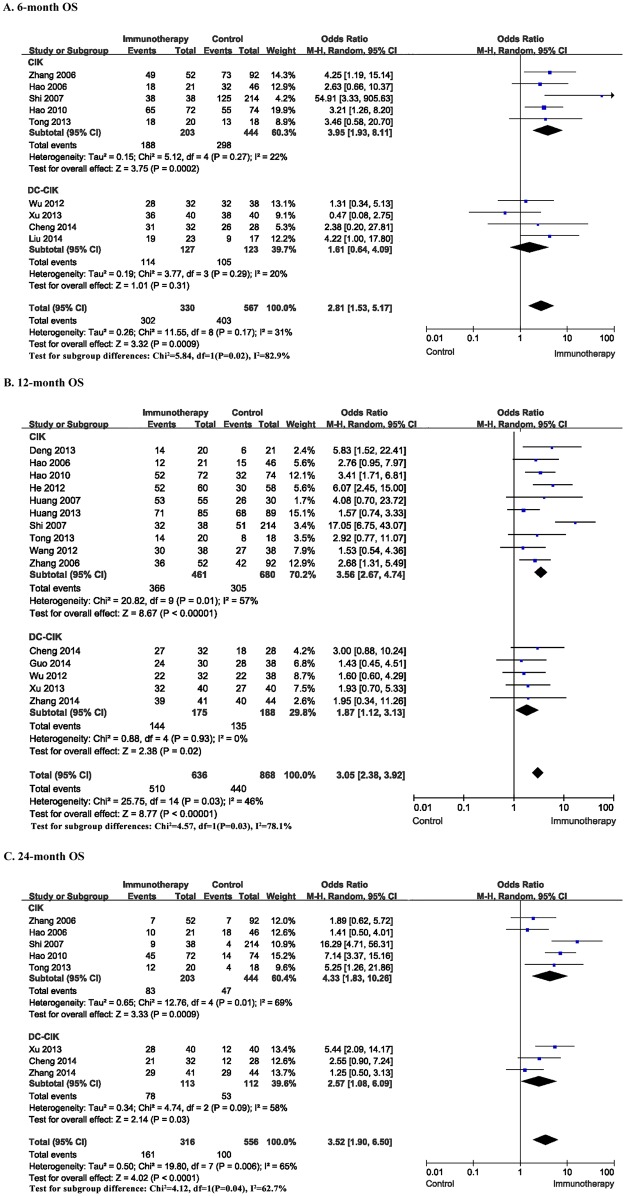
Comparison of 6, 12, 24-month OS between the patients undergoing cellular immunotherapy or not. The random effects model (Mantel-Haenszel method) was used.

Overall survival rate, including 6-, 12-, and 24-month overall survival rate, was significantly higher in HCC patients undergoing cellular immunotherapy, compared to patients in control groups respectively. For 6-month OS, the meta-analysis for five CIK studies and four DC-CIK studies yielded statistically significant differences (OR = 2.81; 95% CI: 1.53–5.17, P = 0.0009). However, there was no statistically significant differences found in the subgroup of DC-CIK studies (OR = 1.61; 95% CI: 0.64–4.09, P = 0.31). For 12-month OS, the meta analysis for ten CIK studies and five DC-CIK studies showed a higher 12-month rate in the patients with the combination therapy (OR = 3.05; 95% CI: 2.38–3.92, P<0.00001). For 24-month OS, the OR of five CIK studies and three DC-CIK studies was significant (OR = 3.52; 95% CI: 1.90–6.50, P<0.0001), suggesting a higher 24-month OS in HCC patients undergoing cellular immunotherapy. Also, in CIK subgroup and DC-CIK subgroup, the 12-month and 24-month OS were both significantly higher in patients with the combination therapy (P<0.05) (Fig 4).

### Assessment of publication bias

**Fig 5:**
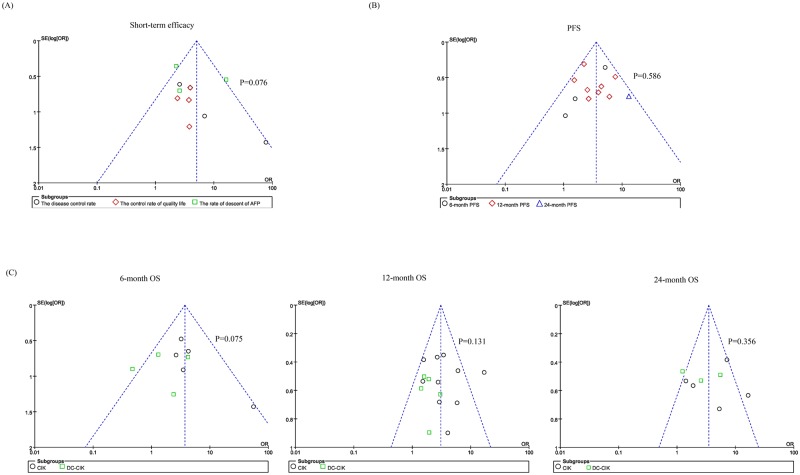
Funnel plots to detect any publication bias. The P value located in this figure indicates the results of Peters test for assessment of publication bias.

In order to guarantee the validity of the conclusions from the meta-analysis, funnel plots and sensitivity analysis were used in subtypes of the short-term efficacy, PFS, and OS (including 6-month, 12-month and 24-month OS). The relative symmetry of all the funnel plots shape suggested the publication bias was not evident. Also, publication bias was also not suggested by Peters test (P > 0.05) (Fig 5).

## Discussion

Our meta-analysis comprehensively analyzed the outcomes of 19 individual studies with 1744 HCC patients from 3 databases, confirming the efficacy of the combination of cellular immunotherapy and TACE-predominant minimally-invasive Treatment on the prognosis of HCC patients.

TACE-predominant minimally-invasive Treatment is one of the main treatments for patients with advanced HCC or patients who are not suitable to surgery [6,39,40]. However, TACE-predominant minimally-invasive Treatment might damage the liver function, reduce the resistance and immunity, and lead to tumor recurrence and residual. So, the combination of TACE-predominant minimally-invasive Treatment and immunotherapy might become an effective treatment [41]. From 18 meta-analysis or reviews in this field, 3 out of 18 performed a meta-analysis on HCC. These systematic reviews focused on the efficacy of the combination of CIK/DC-CIK therapeutic treatment and traditional therapy, respectively. Xie et al [42] studied the efficacy of adoptive immunotherapy in postoperative HCC. Li et al [15] demonstrated that CIK cells transfusion therapy could improve the synergistic effect of HCC patients after TACE or TACE+RFA therapy. Su et al [14] evaluated the efficacy and safety of DC-CIK cell therapy in combination with TACE-predominant minimally-invasive treatment for HCC. However, there was no studies systematically analyzed whether the adjuvant cellular immunotherapy after TACE-predominant minimally-invasive treatment is essential. Notably, it is still controversial that whether CIK or DC-CIK cells immunotherapy was efficacy after different interventional therapy, such as TACE, TACE+RFA, TACE+PEI, and TACE+PMCT for HCC. Our meta-analysis comprehensively analyzed the efficacy of the combination of different cell immunotherapy and different strategy of TACE-predominant minimally-invasive treatment. Through short-term and long-term efficacy analysis, we suggested that the group combined with CIK or DC-CIK cell immunotherapy was associated with better prognosis than minimally invasive treatment alone group.

Our analysis demonstrated that the combination therapy could improve the short-term efficacy, including the disease control rate (P = 0.007), the control rate of quality life (P = 0.003), and the AFP descent rate (P = 0.02). However, the number of included studies for each subgroup was relatively low. And the limitation might lead to bias, even though we found no evidence of publication bias in the meta analysis of the short-term efficacy (P = 0.07) (Fig 5A).

The systematic analysis also showed a significant survival benefit with regard to the PFS and OS when patients undergoing cellular immunotherapy (P≤0.05). There was no heterogeneity across the trails in the meta-analysis for PFS. However, the heterogeneity in 12-month and 24-month OS was statically significant (P<0.05). This demonstrated that the degree of variability was more consistent in each PFS result of study. Notably, for 6-month, 12-month and 24-month OS, the meta analysis was divided into two sub-studies (CIK studies and DC-CIK studies) to reduce the bias caused by different immune cell regimens. For total effect, OS was significantly higher in HCC patients who underwent cellular immunotherapy, compared to patients in control group. However, the heterogeneity in 12-month and 24-month OS in CIK subtypes was statically significant (P<0.05). And the heterogeneity of 6-month, 12-month and 24-month OS between CIK group and DC-CIK group was statically significant (P<0.05) (Fig 4). This result suggested that cellular immunotherapy either CIK or DC-CIK might be needed after different interventional therapy. However, the degree of variability was more consistent in the subtype of DC-CIK studies.

There were some limitations existed in this meta-analysis. First, the number of included studies in each subgroup was relatively low. And the included studies mostly occurred in China lacking multinational larger sample multi-center clinical trials with sufficient statistical power. Second, heterogeneity is a potential issue that may affect the interpretation of the results. The sources of heterogeneity may result from designs and methods, including age distribution, gender, regimens and so on. To address this issue, we employed subgroup analysis and random effects method to perform a meta-analysis on the results of these 19 studies. Third, Publication bias might occurred when the publication of research results depended not just on the quality of the research but on the hypothesis tested, and the significance and direction of effects detected. For meta-analysis, the studies would not be representative when the publication bias presented. In order to guarantee the validity of the conclusions from the meta-analysis, funnel plots and sensitivity analysis (Peters test) were used in the study.

Overall, our systematic analysis demonstrated that the potential clinical value of the combination of cellular immunotherapy with minimally-invasive therapy for prolonging progression free and overall survival. On the basis of the present results, we believe that more advanced and optimized cellular immunotherapy strategies are on their way and may shed a better light on the modality of treatment for HCC.

## Supporting Information

S1 Checklist PRISMA 2009 checklist. (DOC)

S1 Fig The quality assessment for included literatures by QUADAS-2. (TIF)

S2 Fig PRISMA 2009 flow diagram. (DOC)

## Abbreviations

CI: confidence intervals
CIK: cytokine-induced killer
CTL: cytotoxic lymphocyte
DC: dendritic cell
HCC: hepatocellular carcinoma
I^2^: inconsistency index
LAK: lymphokine-activated killer
NK: natural killer cell
OR: odds ratio
OS: overall survival
PFS: progression free survival
QUADAS-2: quality assessment of diagnostic accuracy studies-2
TACE: transcatheter arterial chemoembolization
TIL: tumor infiltrating lymphocyte
