# Supplementary figures and images for: Is Adjuvant Cellular Immunotherapy Essential after TACE-Predominant Minimally-Invasive Treatment for Hepatocellular Carcinoma? A Systematic Meta-Analysis of Studies Including 1774 Patients
Source: PLoS One. 2016 Dec 22;11(12):e0168798. doi: 10.1371/journal.pone.0168798 (PMC5179243; doi:10.1371/journal.pone.0168798)

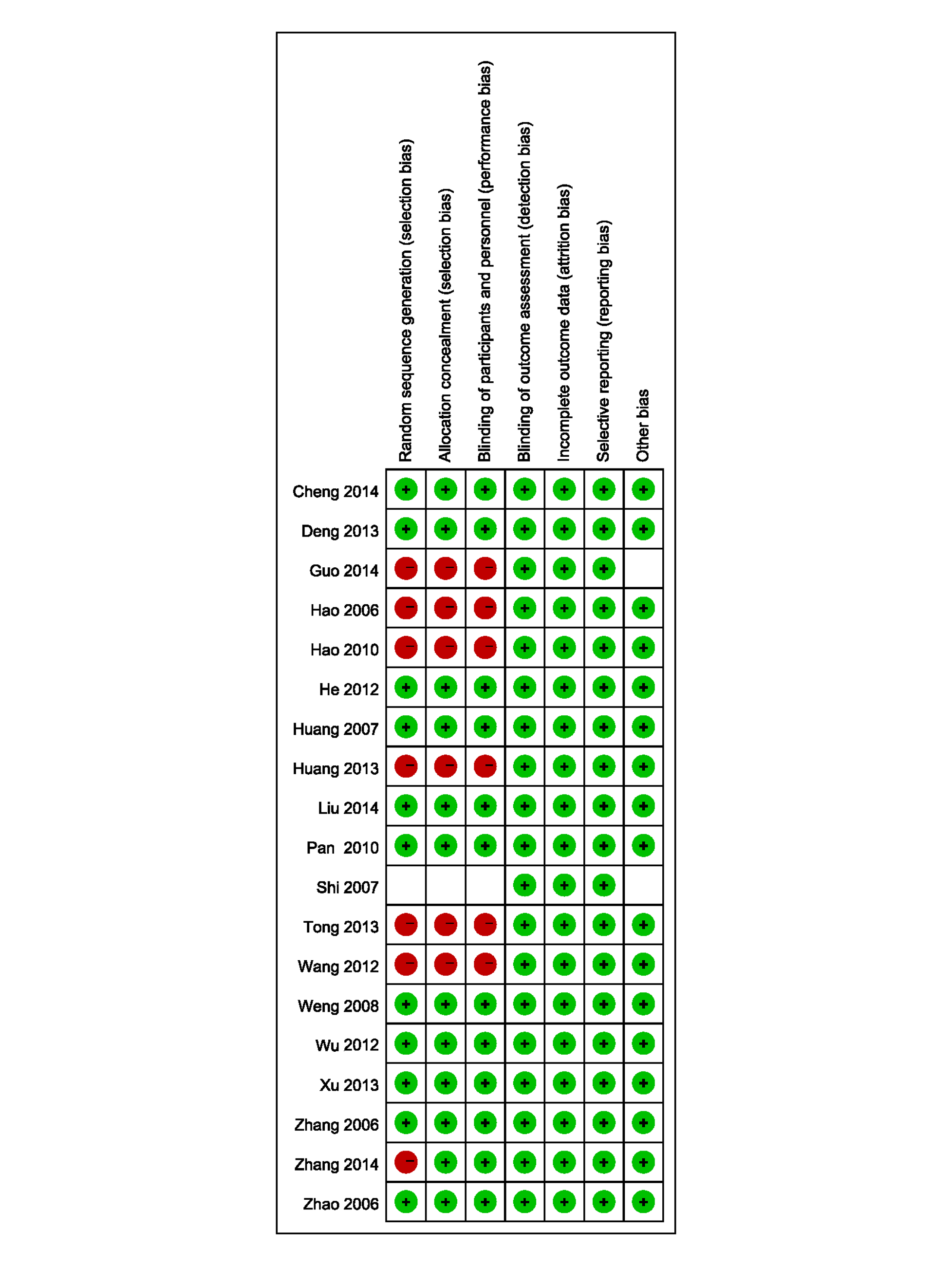

Supplement: S1 Fig — (TIF) [file pone.0168798.s002.tif]
