# Supplementary material for: Is Adjuvant Cellular Immunotherapy Essential after TACE-Predominant Minimally-Invasive Treatment for Hepatocellular Carcinoma? A Systematic Meta-Analysis of Studies Including 1774 Patients
Source: PLoS One. 2016 Dec 22;11(12):e0168798. doi: 10.1371/journal.pone.0168798 (PMC5179243; doi:10.1371/journal.pone.0168798)
Supplement: S2 Fig — (DOC) [file pone.0168798.s003.doc]

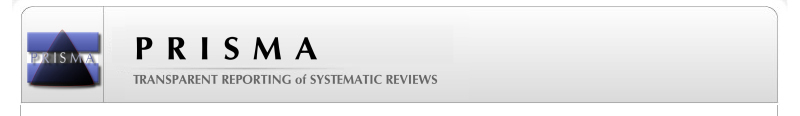
**PRISMA 2009 Flow Diagram**

**Screening**

**Included**

**Eligibility**

**Identification**

Records identified through database searching
(n = 1174 )

Additional records identified through other sources
(n = 18 )

Records after duplicates removed
(n =962 )

Records screened
(n =79 )

Records excluded
(n = 883 )

Full-text articles assessed for eligibility
(n = 19 )

Full-text articles excluded, with reasons
(n = 60 )

Studies included in qualitative synthesis
(n = 19 )

Studies included in quantitative synthesis (meta-analysis)
(n = 19 )
